# The impact of external and internal focus of attention on visual dependence and EEG alpha oscillations during postural control

**DOI:** 10.1186/s12984-022-01059-7

**Published:** 2022-07-26

**Authors:** Lei Ma, Peter J. Marshall, W. Geoffrey Wright

**Affiliations:** 1 Department of Health and Rehabilitation Sciences, Temple University, Pearson Hall, 1800 N Broad St, 19121 Philadelphia, PA USA; 2 Department of Psychology and Neuroscience, Temple University, Weiss Hall, 1701 North 13th St, 19122 Philadelphia, PA USA

## Abstract

**Background:**

The ability to maintain upright posture requires successful integration of multiple sensory inputs (visual, vestibular, and somatosensory). When one or more sensory systems become unreliable, the postural control system must “down-weight” (or reduce the influence of) those senses and rely on other senses to maintain postural stability. As individuals age, their ability to successfully reweight sensory inputs diminishes, leading to increased fall risk. The present study investigates whether manipulating attentional focus can improve the ability to prioritize different sensory inputs for postural control.

**Methods:**

Forty-two healthy adults stood on a balance board while wearing a virtual reality (VR) head-mounted display. The VR environment created a multisensory conflict amongst the different sensory signals as participants were tasked with maintaining postural stability on the balance board. Postural sway and scalp electroencephalography (EEG) were measured to assess visual weighting and cortical activity changes. Participants were randomized into groups that received different instructions on where to focus their attention during the balance task.

**Results:**

Following the instructions to direct attention toward the movement of the board (external focus group) was associated with lower visual weighting and better balance performance than when not given any instructions on attentional focus (control group). Following the instructions to direct attention towards movement of the feet (internal focus group) did not lead to any changes in visual weighting or balance performance. Both external and internal focus groups exhibited increased EEG alpha power (8–13 Hz) activity over the occipital cortex as compared to the control group.

**Conclusions:**

Current results suggest that directing one’s attention externally, away from one’s body, may optimize sensory integration for postural control when visual inputs are incongruent with somatosensory and vestibular inputs. Current findings may be helpful for clinicians and researchers in developing strategies to improve sensorimotor mechanisms for balance.

## Background

Postural control is a complex sensorimotor skill that requires the nervous system to successfully integrate multisensory inputs (visual, vestibular, and somatosensory) in order to maintain upright stability and orientation with the external environment [1]. When the reliability of one or more senses diminish, the brain must rely less on unreliable sensory systems and more on the remaining intact sensory systems to achieve stable, upright balance [2–4]. Degradation of vestibular and somatosensory systems due to normal aging or diseases increases visual dependence during postural maintenance [4–6]. The increased visual dependence is often associated with a greater risk for falls as individuals become unable to reduce their reliance on visual feedback, even when vision is unreliable [2, 6].

One volitional method that can improve postural control involves directing one’s attentional focus to specific stimuli or components of movement [7–12]. Studies have shown that an external focus of attention on the intended effects of one’s movement (i.e., focusing on the outcome or the effects of one’s action) is associated with improved postural control compared to an internal focus of attention on a specific body part during movement [7, 8]. It has been hypothesized that an internal focus disrupts automatic control processes that regulate motor behavior, while an external focus optimizes these control processes and reduces attentional demands [7, 12]. However, recent studies have suggested that internal focus may be beneficial when increased reliance on somatosensory information is needed [9, 11]. Becker and McNamara [9] showed that individuals using internal focus to maintain stability while standing blindfolded on a balancing platform are better at reducing the variability of the platform than when using external focus. Without vision, internal focus may improve postural control by prioritizing somatosensory input for continuous sensory feedback. Nevertheless, it is unclear from kinematics or task performance that there are underlying sensory integration changes. Understanding the neural mechanisms through which attentional focus changes sensory integration can provide further insights into fall prevention.

Sensory integration for postural control can be measured using sensory manipulations to challenge the nervous system. By creating multisensory conflicts during a balance task, the nervous system must change its reliance, or “weighting”, on different sensory inputs (i.e., visual, vestibular, and somatosensory) to perceive orientation and maintain stability [2, 3]. Subtle visual movements of the surrounding environment can influence the estimation of self-motion. During low amplitude, slow oscillation visual stimulation, body sway will follow the frequencies of visual stimulation as the result of the false sense of self-motion [2, 3, 13–15]. This stimulus-response behavior allows us to systematically measure the coupling between visual input and postural control (i.e., visual weighting). Studies have shown that explicit knowledge of visual manipulations can decrease the influence of visual manipulation [13, 14]. This suggests a role for attention in adjusting the visual weighting for postural control. The present study investigates how attentional focus may help resolve multisensory conflict for postural control. More specifically, we assessed visual weighting on postural sway when individuals were exposed to visual manipulations incongruent with somatosensory and vestibular inputs. If an internal focus of attention is better at prioritizing the reliable sensory inputs for balance [9], this may reduce visual weighting.

The present study also explores neural correlates of visual weighting for postural control. Increased alpha power (8–13 Hz) over the occipital cortex has been related to the suppression of irrelevant visual information [16–18]. Changes in frontal alpha power have been suggested to reflect top-down control of visual processing [19, 20]. It is unclear whether these alpha power changes can be identified when the nervous system must reduce reliance on visual input to maintain postural stability. If attentional focus changes visual weighting under the current experimental paradigm, changes in alpha power over the occipital and frontal cortices may also be apparent.

## Methods

### Participants

Forty-two participants (demographic data, see Table 1) were randomly assigned to three groups (*n* = 14 per group): Control (CON), internal focus (INT), and external focus (EXT) groups. Group assignments were determined at the beginning of the study and given sequentially after participants met the inclusion and exclusion criteria. All participants had normal or corrected-to-normal vision. Exclusionary criteria included any neurological or musculoskeletal conditions that limited the ability to stand or walk independently, and/or a self-reported history of vestibular or balance issues. The Temple University Institutional Review Board approved the procedures, and all participants gave informed consent before starting the study. Participants received a $15 gift card upon the completion of the study.

**Table 1:** Demographic data

Group	CON	EXT	INT	p-value
Age (years)	25.6 (4.1)	26.7 (8.5)	25.1 (4.8)	0.773
Height (cm)	174.8 (8.4)	168.2 (11.2)	176.0 (10.5)	0.105
Weight (kg)	74.0 (14.1)	68.1 (12.3)	75.3 (13.7)	0.328
Dominant hand	13R 1 L	13R 1 L	11R 3 L	0.422
Sex	7 F 7 M	7 F 7 M	5 F 9 M	0.697
Self-report of percentage of attention invested at the instructed focus during block 2 (%)	N/A	74.3 (10.9)	82.1 (9.7)	0.054
Number of participants in kinematic analysis	14	14	14	
Number of participants in EEG analysis	13	12	14	

Age, height, weight, percentage of attention invested to the instructed focus are shown in mean with standard deviation in parentheses. Age, height, weight, right-handedness, and female per group were compared among the three groups using a one-way ANOVA. Percentage of attention invested between EXT and INT group was compared using a two-sample t-test

Sample size was calculated based on an *a priori* power analysis for one-way ANCOVA of three groups (G*power) with a single covariate. To test for the null hypothesis of no differences among the three groups, we applied an effect size of 0.5 and an alpha of 0.05 to achieve a power of 0.8. The selected effect size was determined from pilot findings and the meta-analysis by Kim, Jimenez-Diaz, and Chen (2017) on internal versus external focus during balance tasks.

### Visual stimulation

**Fig. 1:**
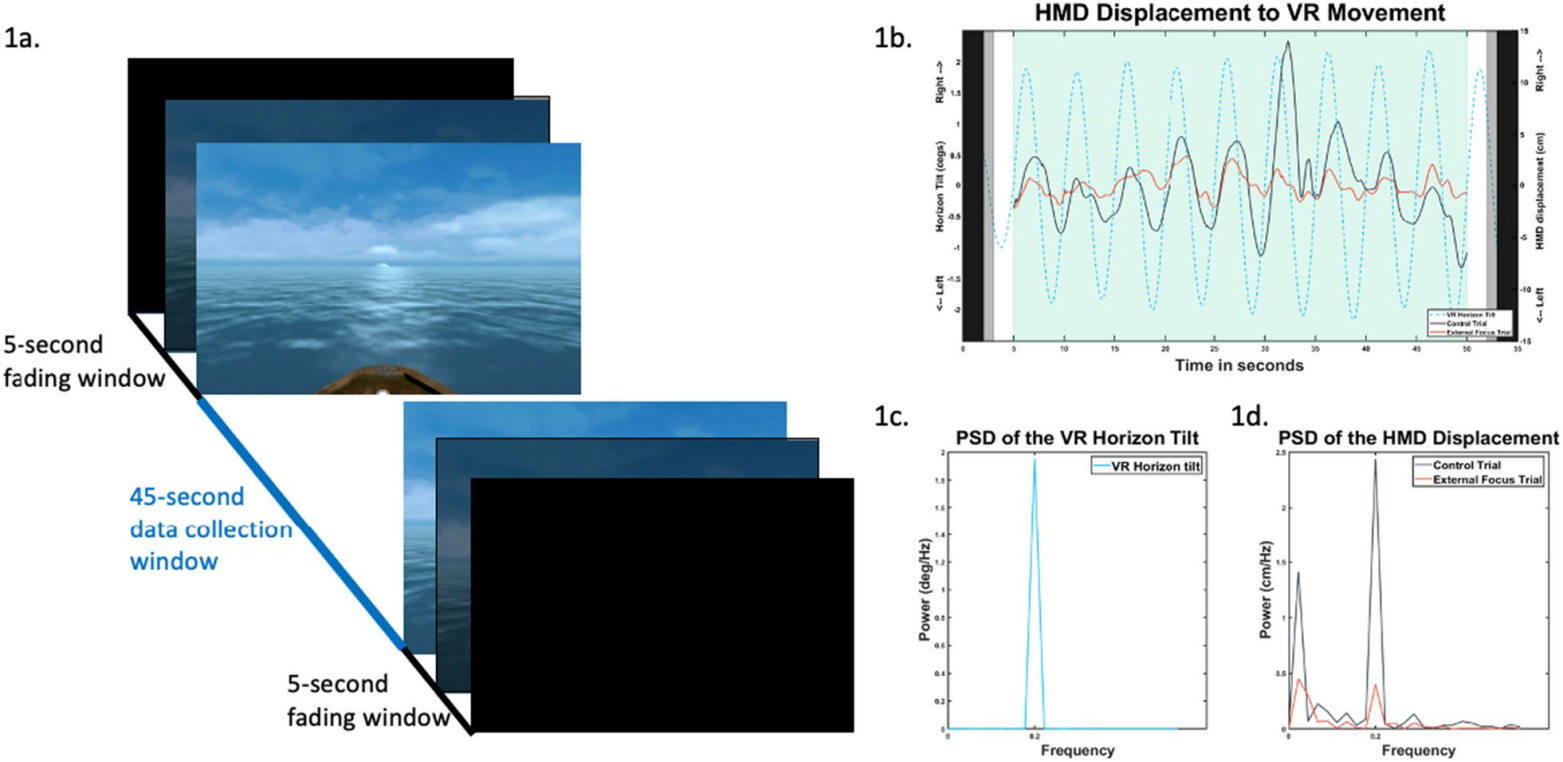
**a** Visual stimulation setup. From initiation to termination, the trial was 55 s. Only the 45-second window between the start and the end of the trial was used for data analysis. **b** Time series example from a single subject. The dashed blue line is an example of the VE horizon motion. The solid black line is an example trial of head ML movement when the participant was given the control instructions. The solid orange line is an example trial of head ML movement when the participant was given the external focus instructions. **c** Power spectral density of the VR horizon time-series data from (**b**). **d** Power spectral density of the head ML movement from control and external focus time-series data in (**b**)

A VR head-mounted display (HMD; Pico Neo 2 Eye, San Francisco, CA) and a rocker board (Blue Planet Balance Surfer, Honolulu, HI) were used to alter visual and somatosensory input, respectively, and perturb postural responses. The HMD provides an immersive visual first-person perspective of standing on a boat on a body of water. The virtual environment was developed in Unity 3D (San Francisco, CA), and the rendering of the water was created using the Water4 Prefab from Unity’s Standard Assets Environment. When looking straight ahead, participants saw the bow of the boat, the horizon over the animated water, and an iceberg at the middle of the horizon (Fig. 1a). The rocker board only tilted in the mediolateral (ML) direction with a maximum tilt angle of 17.55 degrees from the floor. The use of the rocker board was to decrease postural stability by allowing greater body sway. Our setup created a conflict between multiple sensory systems: (1) Visual input from the VR scene indicating that the world is tilting, (2) a mismatch between visual and vestibular inputs in conveying gravitational verticality, and (3) a mismatch between somatosensory input from the rocker board and visual and vestibular inputs regarding body position and orientation.

### Experimental trials

For every trial in the study, the timeline of presentation of the VR scene and the data recording were as follows: An initial 5-second fade-in window, followed by a 45-second data collection window, and finally, a 5-second fade-out window (Fig. 1a). When the experimenter started the trial, the participant initially saw a black screen. The VR scene gradually faded in after a random delay of 2–3 s. The transition from the black scene to the VR scene lasted 1 s. Data collection started 5 s after the start of the trial. After the 45-second recording window, the scene gradually faded back to the black scene. This fading process was initiated at a random time between 2 and 3 s within the 5-second fade-out window (Fig. 1a).

Participants performed all the trials while standing, feet shoulder-width apart on the rocker board, with their arms across their chest, surrounded by a bariatric walker, and monitored by a research assistant. Participants were required to grab onto the walker after each trial. There was a mandatory minimum 30-second break between trials.

### Instructions

Participants first performed two practice trials with no tilting motion of the virtual visual horizon. However, the rendering of the waves in the scene was always active, and participants reported visual motion immediately upon seeing the scene. For the first practice trial, participants were encouraged to visually explore the VR scene to get accustomed to the experimental setup. For the second practice trial, the experimenter explained to the participants that the primary goal for all the trials in this study was to look straight ahead at the iceberg and keep their bodies as still and upright as possible. It was also explained that they might be given additional instructions to help them maintain their stability in the later trials. Participants were asked to explain the instructions back to the experimenter to demonstrate understanding of the task before continuing the experiment.

Within each of the subsequent blocks (i.e., blocks 1 and 2), participants performed three trials with the same VR scene as in the practice trials but with the horizon tilting at randomly generated peak-to-peak amplitudes of 1.8 to 2.2 degrees at the frequency of 0.2 Hz (Fig. 1b). Participants were not told that the horizon would be moving. Based on our preliminary testing, the selected parameters minimized potential habituation to the VR tilt motion and was found to be a subthreshold level of visual perception. Before block 1, all participants were given the control instructions (Table 2). Before block 2, INT group participants received the internal focus instructions, the EXT group participants received external focus instructions, and CON group participants received the control instruction again (Table 2). The instructions were modeled after previous studies on attentional focus for balance tasks [21–23].

**Table 2:** Instructions for each attentional focus group: control group (CON), external group (EXT), and internal group (INT)

Group	Instructions
CON	“The VR scene may or may not move. Regardless of whether it moves or not, please look straight ahead at the iceberg on the horizon and try to keep yourself as still and upright as possible.”
EXT	“In order to help you keep still and upright, please look straight ahead at the iceberg on the horizon and pay attention to how the board is moving. Focus on keeping the rocker board leveled. We will be looking at how well the board is leveled”
INT	“In order to help you keep still and upright, look straight ahead at the iceberg on the horizon and pay attention to how your feet are moving. Focus on keeping both feet leveled to each other. We will be looking how well your feet are leveled.”

After each trial in block 2, the INT and EXT groups were asked to rate the percentage of attention that they directed towards the instructed focus area [23]. If a participant reported investing less than 50% of their attention on the instructed focus, the trial would be repeated without the participant’s knowledge [23]. In the current study, no participant reported investing less than 50% on the instructed focus. Participants in the CON group were not asked to rate their level of attention so as not to induce an attentional strategy or bias attentional focus. The average levels of attention between EXT and INT groups are shown in Table 1.

### Kinematic measures for postural control

The HMD used in this study is embedded with an inertial measurement unit and two cameras that use “inside-out” tracking of the HMD position [24]. We used the ML position data from the HMD to measure lateral stability of the participant during the balancing task [25, 26]. The displacement data was sampled at 50 Hz and filtered with an 8th order Butterworth low-pass filter at 20 Hz cut-off. Dependent variables for balance performance were the root mean square of ML displacement (RMS), root mean square of ML velocity (RMSv), and total path length of ML displacement. These variables are common measures for assessing postural stability (i.e., RMS and total path length) and effort to maintain stability (i.e., RMSv) [26, 27].

We calculated the visual gain response as our primary dependent variable to quantify the visual influence on postural control [2, 14]. To calculate visual gain, we first calculated the frequency response function that measures the strength of the relationship between an input signal *x(t)* and an output signal *y(t)* in the frequency domain (Fig. 1c and d). For each trial, Fourier transforms of *x(t)* and *y(t)* were calculated using the MATLab function fft(). Then the frequency response function *H(f)* was calculated as follows:

1 $$H\left(f\right)=Y\left(f\right) / X\left(f\right)$$

Where *Y(f)* over *X(f)* is the ratio of the fast Fourier transforms of output (i.e., *y(t)* or ML head displacement) over input (i.e., *x(t)* or VR horizon tilt). Then, we evaluated *H(f)* at 0.2 Hz, which is the frequency of VR horizon oscillation. The magnitude (absolute value) of |*H(0.2 Hz)|* is the visual gain response [2, 14, 28]. A higher visual gain indicates a stronger relationship between the visual stimulus and the postural response, i.e., stronger visual influence. A lower visual gain response indicates the opposite, i.e., weaker visual influence.

### EEG alpha power

Continuous EEG was recorded using a 32-channel stretch cap (ANT Neuro, Berlin). Data were sampled at 512 Hz. Scalp electrode impedances were kept below 15 kΩ and referenced to the Cz channel during collection. EEG signals were amplified using optically isolated, high input impedance (> 1 GΩ) custom bioamplifiers (SA Instrumentation, San Diego) and digitized using a 16-bit A/D converter. Bioamplifier gain was 4,000 and the hardware filter (12 dB/octave roll-off) settings were 0.1 Hz (high-pass) and 100 Hz (low-pass).

The EEGLAB toolbox for MATLAB was used for offline EEG processing. A zero-phase finite impulse response notch filter was applied with cut-offs at 59 and 61 Hz to remove electrical line noise. The data was then bandpassed between 1.5 and 100 Hz using the zero-phase FIR filter [29]. Noisy channels were identified using the pop_clean_rawdata() plugin function for EEGLAB. Channels that were (1) flat for more than 5 s, (2) showed high-frequency noise values over four standard deviations, or (3) correlated with nearby channels by less than 0.6 were removed [30]. On average 1.8 channels were removed per participant during this step. Data were then re-referenced to the common average reference, and the removed channels were then interpolated using the spherical spline method. Each participant’s EEG data were epoched at 45 s per trial, 5 s after the starting trigger, and 5 s before the end of the trial (Fig. 1a). An extended Independent Components Analysis was then performed on each participant’s epoched data using the default parameters in EEGLAB [31].

**Fig. 2:**
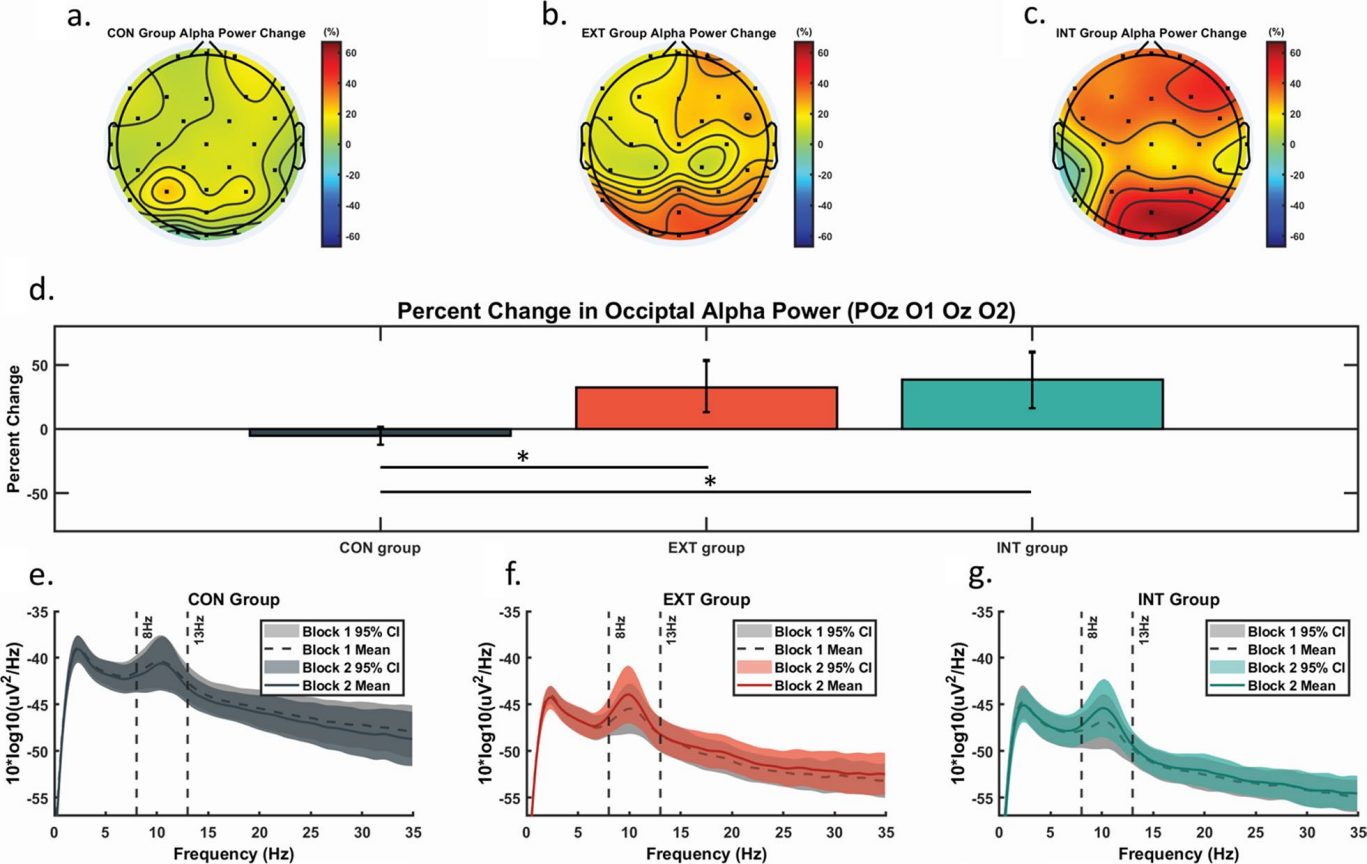
**a**, **b,** **c** Topographical plot of the scalp EEG channels for mean percent change in alpha power (8–13 Hz) from block 1 to block 2 for each of the three groups. **d** Mean percent change (95% confidence interval) of occipital alpha power among the three experimental groups. Control group (CON) in dark grey, external group (EXT) in orange, and internal group (INT) in teal. *Post-hoc tests reveal a significant difference (p < 0.05) between EXT vs. CON and INT vs. CON. Error bars are in 95% confidence interval. **e**, **f**, **g** Absolute power frequency for each of the groups at block 1 and 2. Dashed lines represent mean power at block 1. Solid lines represent mean power at block 2. The shaded area represents 95% confidence interval, with light grey representing block 1’s 95% confidence interval for each group

The ICLabel function in EEGLAB was used to identify artifactual Independent Components (ICs) automatically [32]. These include ICs with eye blinks, muscle activity, line noise, and channel noise. To maintain reproducibility and prevent subjective bias, ICs identified by ICLabel to be over 80% probability for those artifactual IC were automatically removed. On average, 5.6 ICs were removed. The power spectral density for each channel was then calculated using the Welch method with Hamming windows of 1 s and 50% overlap. The power density between frequencies of 8 to 13 Hz was first mean averaged per channel to measure alpha power. Alpha power from channels POz, O1, Oz, and O2 was mean averaged to assess occipital alpha power. Alpha power from channels F3, Fz, and F4 was averaged to assess frontal alpha power. The selection of these channels for analysis was based on the observed changes in alpha power topography among the three groups (Fig. 2a–c). Three (2 EXT and 1 CON) participants’ data were excluded from analysis due to technical issues during data collection or excessive channels removed by pop_clean_rawdata() function (i.e., > 4 channels).

### Statistical analysis

The primary aim of the study was to examine the differences in visual weighting among the three attentional focus groups (CON, INT, and EXT). One-way analysis of covariance (ANCOVA) was conducted to test for differences among the three groups in visual gain for block 2, with visual gain from block 1 set as the covariate. In addition, performance outcomes at block 2 as measured by RMS, RMSv, total path length were also analyzed using ANCOVA, with their respective scores at block 1 set as the covariate. If ANCOVA revealed significance, Bonferroni corrected pairwise comparisons were used in post-hoc analysis. Pearson correlations were performed to assess the relationship between the score change (block 2 – block 1) in visual gain and the change in each of the three balance performance variables across all participants regardless of the groups.

The secondary aim of the study was to determine whether different attentional focuses would lead to observable alpha power changes and whether alpha power changes correlate to visual gain and performance changes. We normalized the EEG data by taking the percent change from block 1 to block 2 due to high variability in alpha power across individuals [33] and accounting for baseline cortical differences from the current balance task. We applied Welch’s analysis of variance (ANOVA) to measure the percentage change scores across the three groups for the alpha power percent change at the occipital and frontal channels due to unequal variance detected by Levene’s Test. If Welch’s ANOVA revealed significance, the Games-Howell test was performed for post-hoc comparison. Pearson correlations were performed to assess the relations between percent change in alpha power and percent change in visual gain and performance outcomes across all participants. Statistical analyses were performed using IBM SPSS (version 27) with a significance level set at 0.05.

## Results

### Visual gain and performance measures

The Levene’s tests revealed that the variances were equal for visual gain response (F(2,39) = 0.07, p = 0.901), RMS (F(2,39) = 3.024, p = 0.060), RMSv (F(2,39) = 1.419, p = 0.254), and total path length (F(2,39), p = 0.300) at block 2. We visually inspected the regression lines for each of the dependent variables and its covariate across group for linearity. Additionally, homogeneity of regression slopes was assessed by conducting a covariate by group interaction analysis for each of the dependent variables using an ANCOVA model. This analysis revealed non-significant covariate by treatment interactions for the visual gain response (F(2,36), p = 0.288), RMS (F(2,36) = 0.718, p = 0.495), RMSv (F(2,36) = 1.377, p = 0.265), and path length (F(2,36) = 1.460, p = 0.246).

**Fig. 3:**
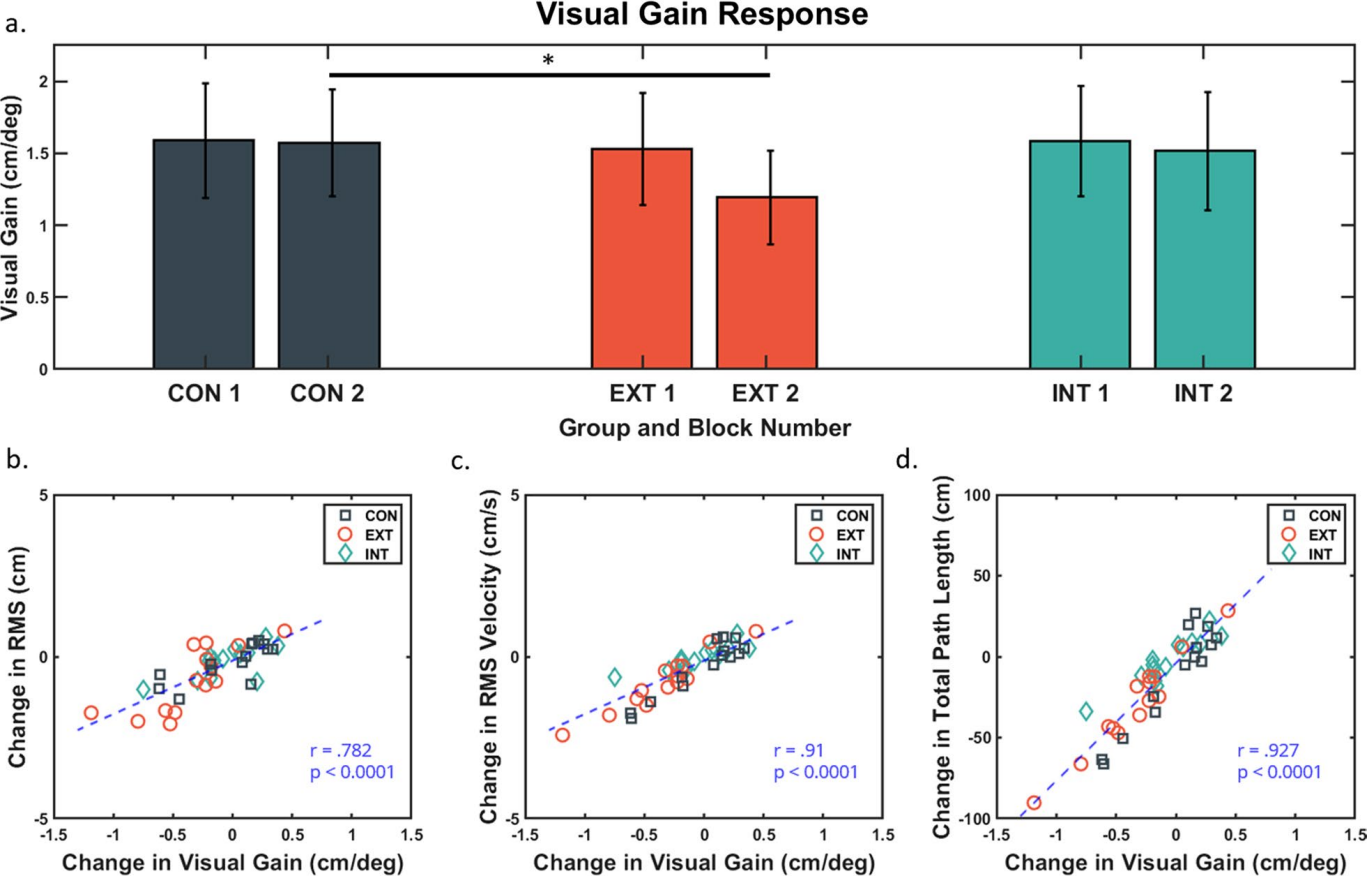
**a** Mean visual gain scores at block 1 and 2 for all three groups. Control group (CON) in dark grey squares, external group (EXT) in orange circles, and internal group (INT) in teal diamonds. Error bars are in 95% confidence interval. Significant difference between EXT and CON group after Bonferroni correction and covariate adjustment. **b**, **c**, **d** Scatterplot showing the relationship between visual gain scores to root mean square (RMS) of mediolateral (ML) headset displacement, RMS of ML headset velocity, and total path length of ML headset displacement, respectively

There was a significant difference in visual gain among the three groups at block 2 (F(2,38) = 4.302, p = 0.021, η_p_^2^ = 0.185) after accounting for visual gain scores at block 1. Post-hoc Bonferroni adjusted comparisons revealed that EXT group was significantly lower than the CON group for visual gain response (p = 0.029). INT versus EXT and INT versus CON did not reach significance (Fig. 3a).

There was a significant difference in RMSv (F(2,38) = 4.17, p = 0.023, η_p_^2^ = 0.18) and total path length (F(2,38) = 4.476, p = 0.018, η_p_^2^ = 0.191) among the three groups at block 2 after accounting for block 1 scores. Post-hoc Bonferroni adjusted comparisons revealed the EXT group to be significantly lower than the INT group for RMSv (p = 0.023) and total path length (p = 0.018). EXT versus CON and INT versus CON did not differ significantly. There was no significant difference in RMS among the groups at block 2 (F(2,38) = 1.062, p = 0.089, η_p_^2^ = 0.12), after controlling for block 1 RMS scores.

Pearson correlations on score changes (block 2 – block 1) from all participants revealed significant relationships between change in visual gain vs. change in RMS (r = 0.782, p < 0.0001), versus change in RMSv (r = 0.910, p < 0.001), and versus change in total path (r = 0.927, p < 0.0001) (Fig. 3b–d).

### Occipital alpha power

Levene’s Test for occipital alpha power revealed unequal variance across the three groups (F(2,36) = 8.486, p = 0.001). Therefore Welch’ ANOVA for unequal variance was performed. Welch’s ANOVA showed a significant difference among groups for percent change in occipital alpha power (F(2, 18.97) = 10.375, p = 0.001). Post-hoc Games-Howell tests revealed that percent change in the EXT was significantly higher than CON (p = 0.014) and INT was significantly higher than CON (p = 0.007). EXT versus INT did not differ significantly (Fig. 2d).

Pearson correlations on the percent change of scores from all participants did not reach significance for occipital alpha power versus visual gain (r = − 0.315, p = 0.05), versus RMS (r = − 0.312, p = 0.054), versus RMSv (r = − 0.182, p = 0.269), or versus total path length (*ρ* = − 0.235, p = 0.149).

### Frontal alpha power

**Fig. 4:**
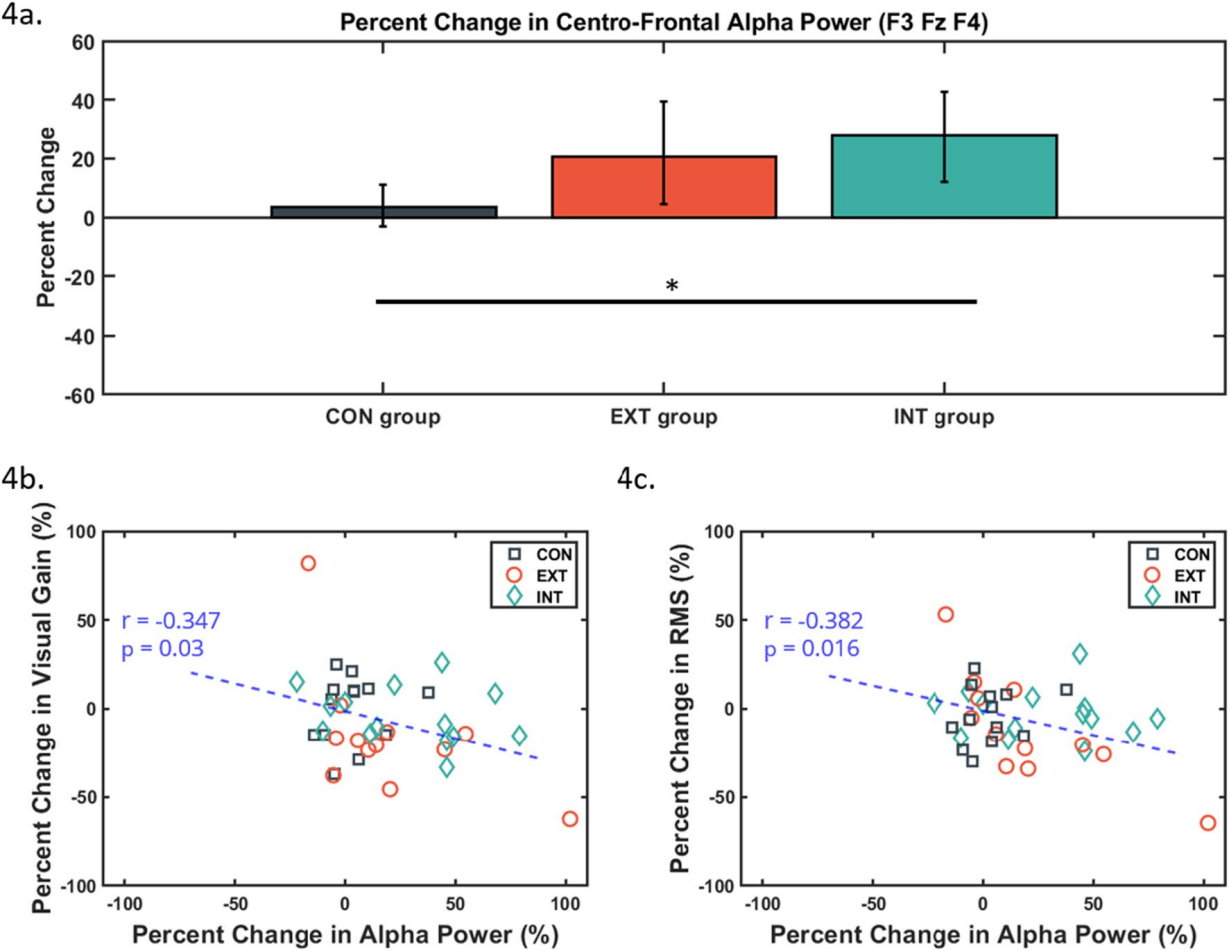
**a** Mean percent change of frontal alpha power among the three experimental groups. Control group (CON) in dark grey squares, external group (EXT) in orange circles, and internal group (INT) in teal diamonds. *Post-hoc Games-Howell tests reveal a significant difference (p < 0.05) between INT vs. CON. Error bars are in 95% confidence interval. **b**, **c**. Scatterplot showing the relationship between frontal alpha power percent change to visual gain and root mean square (RMS) of mediolateral (ML) headset displacement

Levene’s Test for frontal alpha power percent change revealed unequal variance within the three groups (F(2,36) = 8.486, p = 0.001). Therefore Welch’ ANOVA for unequal variance was performed. Welch’s ANOVA showed a significant difference among the groups for percent change in frontal alpha power (F(2, 20.16) = 4.162, p = 0.031). Post-hoc Games-Howell tests revealed that the percent change in the INT group was significantly higher than CON (p = 0.041) (Fig. 4a). EXT versus INT and EXT versus CON did not differ significantly.

Pearson correlations on the percent change of scores from all participants revealed a significant relationship between frontal alpha power percent change versus visual gain (r = − 0.347, p = 0.03) and versus RMS (r = − 0.382, p = 0.016) (Fig. 4b,c). However, RMSv (r = − 0.256, p = 0.115) and total path length (r = − 0.307, p = 0.057) did not reach significance.

## Discussion

A primary goal of the current study was to investigate whether external and internal attentional focus could improve sensory integration when the postural control system needs to resolve a multisensory conflict. When vision is removed or becomes unreliable, precise interpretation of somatosensory and vestibular inputs is critical for the nervous system to estimate how the body moves. Becker and McNamara [9] showed that internal focus on the feet improved postural control when their participants were blindfolded while standing on a rocker board. It is possible that in their study, internal focus may have optimized somatosensory processing for body orientation and postural stabilization, but this was in the absence of any visual input. In the current study, rather than removing vision completely, the participants were exposed to visual manipulations that created a false sense of self-motion. To stabilize balance, the participants needed to down-weight the non-veridical visual input because it was incongruent with somatosensory and vestibular inputs. We hypothesized that if internal focus prioritized somatosensory input for balance, then there may be a concomitant decrease in visual influence on postural sway. However, current results reveal that the internal focus group did not have a lower visual weighting when compared to external focus or control. Conversely, the external focus group demonstrated a significantly lower visual gain when compared to the control group, suggesting that external focus may better optimize the reweighting of non-veridical visual inputs. One potential explanation for the lower visual gain response observed in the external focus group is that attention to the board may have altered spatial awareness of the body and the board. Studies investigating the use of external devices to perform a task (i.e. “tool-usage”) have shown that attention to the external tool can alter the somatosensory representation of the body and spatial perception of the external world [34–36]. While there is limited evidence on how individuals incorporate rocker boards as a tool, we speculate that directed focus on the board may have led to a change in spatial awareness that lowered visual weighting when compared to the other conditions. Because we did not directly measure changes in somatosensory or vestibular weighting, even if attentional focus led to a down-weighting of visual inputs, it is not apparent in our data that a compensatory up-weighting of somatosensory or vestibular inputs occurred.

There are several differences in the current study protocol compared to previous studies, which may have contributed to variations in outcomes [8]. The first relates to how the interpretation of the instructions may have affected postural control. The intent of the external focus instructions differs from previous balance studies on attentional focus. Specifically, other studies used additional feedback, such as controlling a laser pointer or holding onto a balance pole/baton to guide their external focus [37–39], which also changed the goal of the task. Another difference relates to visual feedback. To ensure that the visual input did not provide any feedback or cue regarding body position or board movement, we used a VR HMD to create an immersive visual experience. By only allowing intrinsic feedback, all the groups were afforded the same somatosensory inputs to orient where they and the board were. Thus, the observed differences between groups resulted from a change in attention rather than a change in the task goal or visual feedback of body and board position.

It is possible that due to the instructed attentional focus participants may have adopted new or adapted current motor strategies, such as changes in anticipatory postural adjustments or muscle activations [11, 40, 41]. A previous study by Richer et al. (2017) investigating attentional focus during a standing balance task revealed no statistically significant differences in ankle muscle activation when participants adapt to internal versus external focus [23]. Moreover, it has been theorized under an optimal control model that there may be no functional advantage of increasing muscle activations when the RMS of the center of mass is at low magnitudes [42, 43]. In our study, where RMS of head displacement was generally between 2 and 6 cm, we did not expect changes in muscle activations that would result in a shift in motor strategy. Pearson correlations between visual gain and postural sway variables, such as RMSv and total path length, revealed strong associations (r > 0.9) regardless of the groups, with the relationship indicating that individuals with lower visual gain are generally producing less movement. Considering that the goal of the experimental task was to be “as still and upright” as possible, less total movement may be a good indicator of the current task performance. The current performance measure findings are consistent with previous reports [7, 8] in that external focus led to better balance performance (i.e., maintaining stability on the rocker board).

Increased occipital EEG alpha power has been associated with the suppression of irrelevant visual information [16–19]. In the current study, both external and internal focus groups demonstrated increased occipital alpha power (Fig. 3d). However, there was no difference between these two groups, nor was there a significant correlation between the percent change in occipital alpha power to either visual weighting or balance performance. Given that external and internal focus may affect different regions or pathways in the visual cortex, the channel-level analyses and the low-density EEG array we used may have limited our ability to detect differences between the groups. However, the fact that the control group showed less occipital alpha power compared to internal and external focus groups suggests that both of the instructional groups directed attention away from visual environment. While during control conditions, the goal of keeping “yourself as still and upright as possible” may not direct attention towards intrinsic feedback enough to elicit a decrease in cortical visual processing. Current observations further emphasize the importance of appropriate instructions or cueing to alter cortical processing for postural control.

Frontal alpha power has been suggested to indicate top-down control of visual processing [19, 20]. Changes in occipital alpha power have been theorized to be the product of inhibition-disinhibition mechanisms from the frontal cortex [19]. Current results reveal that frontal alpha power is significantly higher in the internal group than in the control group (Fig. 4a), and that increased alpha power may be associated with decreased visual gain and postural sway variability (Fig. 4b and c). Whether the modulation of visual information is effective may be task-dependent [9] and requires more investigation. In the current study, the external focus group demonstrated improved balance performance and lower visual weighting, suggesting that external focus was more effective in suppressing visual incongruence than internal focus. However, this was not substantiated by changes in frontal alpha power in the external group as they did not differ from the internal or control groups. Nonetheless, the internal focus group exhibited higher frontal alpha power than the control group. Similar to previous studies that theorize that internal focus may disrupt central processes for motor control [7, 12, 44], we speculate that internal focus may have nullified the optimization of sensory inputs for postural control under the current experimental task. However, only a few participants may be driving the correlation between percent change in visual weighting and RMS to frontal alpha power, making the interpretation of the correlation difficult (Fig. 4b and c). The quality of EEG data is also heavily dependent on the filtering parameters and the IC identification and removal at post-processing [29]. The low number of EEG channels in the current study may have limited the spatial resolution and noise removal from IC identification. Future studies using source level analyses [30, 45] from high-density EEG during upright balance may reveal clearer cortical changes from attentional focus.

In terms of other limitations to the current experimental setup, only head displacement was measured. This limited our ability to assess kinematic patterns at other body segments, e.g., hips and ankles. We approached our analysis of postural control using the single-link inverted pendulum model, which is commonly used for postural assessment and has been shown to be functionally capable of assessing postural oscillations [46]. Moreover, upright balance tasks using HMD (e.g., tandem stance, standing on a foam pad) have shown a high correlation between HMDs and center of mass and center of pressure data [25, 47]. Postural sway observed in the current experiment occurs at low amplitudes and slow oscillations and is fixed to the rocker board, providing a window to examine the coupling between vision and postural sway. Another limitation is that the VR scene may have introduced an external cue that confounded the participant’s focus of attention. The iceberg was included as a visual target to help control for the potential influence of gaze on postural control and minimize excess head movements that might occur when visually exploring the scene. Regardless of the assigned group, all participants were told to direct their gaze toward the iceberg at the center of the VR scene to maintain consistency across the groups. Additionally, the control group was told to “keep yourself” as still as possible across all trials, which may have biased attention inwardly. While the general instructions were carefully worded to avoid drawing attention to the body, maintaining balance inherently requires some level of body awareness. The control group may have incorporated internal focus strategies by default from just performing the task. We attempted to control these confounds by giving everyone the same control instructions at baseline (block 1) and using an ANCOVA model to assess differences among the groups after receiving additional instructions (or no additional instruction, i.e. control group) to account for differences at baseline. This approach allowed us to direct our comparisons at differences between the external focus of the board and the internal focus of the feet. The current study cannot also rule out that internal focus, in general, is ineffective in resolving sensory conflicts. It is possible that internal focus on other specific body parts, such as a focus on head or trunk movements, may have led to different results. Further studies are needed to elucidate the effects of attentional focus on sensory integration.

## Conclusions

Age-related declines in visual, vestibular, and somatosensory acuity impact posture and increase the risk for falls in the aging population. By studying how attention can help resolve sensorimotor incongruence in postural control, we gain insight into the interaction between volitional and automatic processes controlling balance. Here we show immediate changes in occipital cortex activity as individuals use different attentional placements when exposed to VR manipulation, which provides a false sense of body position and orientation. However, only attention directed externally from the body helped reduce inappropriate visual influence on postural control. Findings from this study may help guide clinicians and researchers on how best to provide instructions for balance tasks and help an individual develop compensatory strategies to improve balance and prevent falls.

## Data Availability

The datasets used and/or analyzed in the current study are available from the corresponding author on reasonable request.

## Abbreviations

ANCOVA: Analysis of covariance
ANOVA: Analysis of variance
CON: Control group
EEG: Electroencephalography
EXT: External focus group
HMD: Head-mounted display
ML: Mediolateral
IC: Independent components
INT: Internal focus group
RMS: Root mean square
RMSv: Root mean square velocity
VR: V irtual reality
